# Strain-level bacterial identification by CeO_2_-catalyzed MALDI-TOF MS fatty acid analysis and comparison to commercial protein-based methods

**DOI:** 10.1038/srep10470

**Published:** 2015-07-20

**Authors:** C. R. Cox, K. R. Jensen, N. R. Saichek, K. J. Voorhees

**Affiliations:** 1 Department of Chemistry and Geochemistry, Colorado School of Mines, Golden, CO 80401

## Abstract

Matrix-assisted laser desorption/ionization time-of-flight mass spectrometry (MALDI-TOF MS) has emerged as a rapid approach for clinical bacterial identification. However, current protein-based commercial bacterial ID methods fall short when differentiating closely related species/strains. To address this shortcoming, we employed CeO_2_-catalyzed fragmentation of lipids to produce fatty acids using the energy inherent to the MALDI laser as a novel alternative to protein profiling. Fatty acid profiles collected from *Enterobacteriaceae*, *Acinetobacter*, and *Listeria* using CeO_2_-catalyzed metal oxide laser ionization (MOLI MS), processed by principal component analysis, and validated by leave–one-out cross-validation (CV), showed 100% correct classification at the species level and 98% at the strain level. In comparison, protein profile data from the same bacteria yielded 32%, 54% and 67% mean species-level accuracy using two MALDI-TOF MS platforms, respectively. In addition, several pathogens were misidentified by protein profiling as non-pathogens and vice versa. These results suggest novel CeO_2_-catalyzed lipid fragmentation readily produced (i) taxonomically tractable fatty acid profiles by MOLI MS, (ii) highly accurate bacterial classification and (iii) consistent strain-level ID for bacteria that were routinely misidentified by protein-based methods.

Protein-based diagnostic bacterial identification by matrix-assisted laser desorption/ionization time-of-flight mass spectrometry (MALDI-TOF MS) has gained acceptance by the clinical and research communities following U.S. FDA and European Commission CE Mark approval of two commercial systems: the Bruker Microflex Biotyper and the bioMérieux VITEK MS. However, an underappreciated drawback exists when using protein profiling to differentiate between closely related bacterial species or strains, and, as we show in this report, this method often failed to properly differentiate several clinically important species. These ambiguities are thought to arise primarily because closely related species and/or strains express many similar if not identical proteins, but can also occur because of a lack of representative spectra in the manufacturer’s database. To address the commonly held assumption that bacterial diagnostics must target proteins, we investigated fatty acid (FA) profiling by CeO_2_-catalyzed metal oxide laser ionization (MOLI) MS as an alternative means of bacterial ID. By focusing on bacterial lipids as diagnostic biomarkers rather than proteins, and by exploiting the unusual catalytic propensity of the rare-earth lanthanide CeO_2_ to cleaves lipids into FAs as a novel MALDI matrix, we obtained highly accurate and reproducible species- and strain-specific bacterial FA profiles. We hypothesize that this was achieved through rapid, *in situ* conversion of bacterial lipids into FA anions by the 4+ reactive state of cerium using the laser energy inherent to MALDI-TOF MS. Rare earth elements have played an increasingly important role in numerous industries because of their unusual catalytic, electronic, and magnetic properties^1^. Of specific interest, cerium has been increasingly utilized in a wide range of applications including fuel and solar cell construction^2^,^3^, automobile catalytic converters^4^, and as a large-scale catalyst in the hydrogen production and biofuel refining industries^5^. The majority of lanthanides usually exist in a trivalent state^6^. Cerium can exist in either the +3 or +4 valence state, making it an exceptional catalyst with myriad enzymatic mimetic properties^7^,^8^. This dynamic reactivity is thought to occur because cerium has two partially filled electron subshells, 4f and 5d, which allows it to shift between valence states^9^. While cerium has been used in a range of biomedical applications^8^, to our knowledge, its capacity as a biocatalyst for *in situ* conversion of bacterial lipids into taxonomically viable FAs using MALDI-TOF is novel. In previous work, we investigated catalyst stability and reproducibility of CeO_2_-catalyzed MOLI MS analysis with biological replicates of 10 representative bacteria over a 3-week period^6^. ANOVA data clearly demonstrated catalyst stability and showed that a combination of biological and technical sample preparation had no effect on reproducibility. MOLI MS allowed high-throughput strain-level ID of bacterial samples using MALDI instruments already in use in clinical laboratories for protein-based ID. Here we demonstrate the catalytic capability of CeO_2_ applied to rapid ID compared to protein profiling, and provide compelling evidence for its use in accurate bacterial ID. Our protein profiling results obtained on two independent, similarly tuned instruments suggest that *in situ* MOLI MS analysis of FAs can be used alone or as a complimentary technique with existing Biotyper instruments with negative-ion capabilities.

## Results

### CeO_2_-catalyzed MOLI MS fatty acid profiling

**Figure 1:**
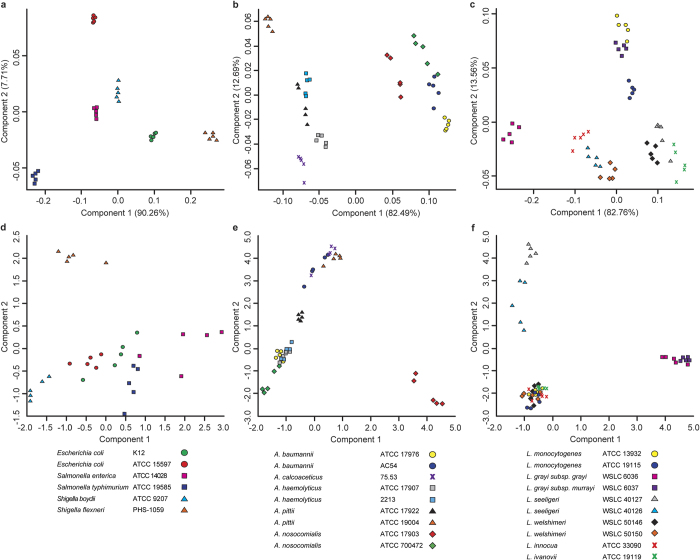
Comparison of protein and CeO_2_-catalyzed fatty acid bacterial identification. PCA plots of CeO_2_-catalyzed fatty acid profiles (**a**) *Enterobacteriacae*, (**b**) *Acinetobacter*, and (**c**) *Listeria*. (**d**-**f**) PCA plots of protein spectra of same phylotypes. The percent variance for each FA component is shown in parentheses.

Because of our early successes using metal oxides for lipid analysis^6^,^10^,^11^,^12^, bacterial genera that were known to be problematic for protein profling^13^,^14^,^15^ were investigated using MOLI MS with CeO_2_ as an alternative means of analysis. Specifically, isolates of three representative sets of clinically-relevant genera belonging to *Enterobacteriaceae*, *Acinetobacter*, and *Listeria* comprised of five replicates each of 26 strains were analyzed and compared with protein-based IDs obtained on two separate Bruker MALDI-TOF instruments (Ultraflextreme and Microflex, both running Biotyper 3.0). In this and previous studies^6^,^16^,^17^, multivariate statistical methods were used to visualize and validate patterns in complex mass spectral data, including bacterial FAs. Score plots from principal component analysis (PCA) of CeO_2_-catalyzed MOLI FA spectra (Fig. 1 top row) and Biotyper protein spectra (Fig. 1 bottom row) for *Enterobacteriaceae, Acinetobacter* and *Listeria* are shown. Color-coded points represent replicates of each bacterial type. Good sample discrimination was indicated by small spatial proximity of individual replicates (inner variance) of one species or strain compared to large distances between clusters of the other species and strains (outer variance). CeO_2_-catalyzed MOLI MS data showed distinct clustering of replicates and observable separation between categories, which indicated that FA profiles for each strain were unique and distinguishable within the species examined in this study. In contrast, and in agreement with Biotyper ID reports (Tables 1, 2, 3 and Supplementary tables 1-3), PCA plots of corresponding protein spectral data showed limited separation and poor clustering, even at the genus level. In some cases poor precision of replicate protein spectral data was also apparent.

Leave-one-out cross-validation (CV) by linear discriminant analysis was used to validate FA data, however, lack of functionality in the Biotyper software prevented similar protein data analysis (the user was simply presented with an automated table of alphanumeric classifications accompanied by corresponding numerical score values). Cross-validation is a predictive statistical modeling technique for determination of class membership^18^. It has been used extensively to validate complex datasets of mass spectra^19^. As shown in Supplementary Tables 4-6, CeO_2_ data was cross-validated to within 98% strain-level accuracy. Correct strain-level ID CV was indicated by the numerical probabilities (high value = high probability) shown in grey for five replicates of each species. The only incorrect classification occurred at the strain level with one replicate of *A. baumannii* AC54 classified as the other *A. baumannii* strain. All replicates were validated to 100% at the species level.

### Biotyper protein profiling

Protein-based Biotyper analysis of the same strains was conducted at the same time as FA-based MOLI MS on two separate MALDI instruments (Bruker Ultraflextreme and Microflex). Protein results were reported as both alphanumeric results and numerical scores assigned to each sample by Bruker’s automated Real-Time Classification software. These scores were based on a comparison of experimentally obtained spectra to Bruker’s Biotyper-specific database containing over 3000 profiles. Scores in the range of 2.300-3.000 provided a highly probable species ID. Scores of 2.000-2.299 indicated a “secure genus ID and probable species ID”; results in the range of 1.700-1.999 were classified as a probable genus ID and scores below 1.699 represented non-reliable ID. All species were also analyzed using an ethanol/formic acid/acetonitrile pre-analysis extraction as suggested by the manufacturer for instances where ID was difficult or failed to yield results^20^. We did not observe an improvement over conventional whole cell sample preparation. Identities of all archived species used in this study were confirmed by 16S rRNA sequencing using universal primers that ensured coverage of all 9 variable regions of the 16s rRNA gene, as previously described^21^,^22^,^23^. However, *A. pittii* and *A. nosocomialis*, were only identified at the genus level and were thus cultured from newly obtained ATCC stocks and analyzed without passage.

### *Enterobacteriaceae* ID

Protein profiling was conducted on five identical colonies of two strains each of *E. coli*, *Salmonella*, and *Shigella*. Results are summarized in Table 1 and are presented as automatically generated in the Biotyper output reports. Instances where Biotyper-derived alphanumeric results were not supported by concomitant numerical score values are indicated in grey with a brief explanation of each discrepancy in parentheses. Two strains of *E. coli* were correctly identified by both protein profiling platforms. All *Salmonella* numerical score values indicated species ID, however, the user was prompted by the Biotyper with a warning indicating these samples could only be accurately typed to the genus level. Both *Shigella* species were misidentified as *E. coli.*

Confirmation of Ultraflextreme results was obtained for each species with analysis on a Microflex Biotyper (Supplementary Tables 1-3). The Ultraflextreme is a more sophisticated instrument that was detuned as described in the Methods section by Bruker service engineers to match Microflex FDA-approved specifications for running the Biotyper software package. While results were similar, some variation was observed across instrument platforms. Of concern, numerous examples in Tables 1, 2, 3 and Supplementary Tables 1-3 demonstrate the tendency for the Biotyper to report alphanumeric species IDs that are supported only by genus numeric score values. Such outcomes can most certainly result in confusion on the part of clinical technicians and lead to the need for added time and cost associated with secondary tests.

### *Acinetobacter* ID

Analyses of two separate strains of five different *Acinetobacter* species were conducted on both instruments. Results are summarized in Table 2 and Supplementary Table 2. *A. baumannii* ATCC 17976 was either misidentified or no reliable ID was provided. *A. baumannii* AC54 was identified to the species by Ultraflextreme analysis, while Microflex analysis gave genus level numeric score values with alphanumeric species ID. *A. calcoaceticus* 75.73 was misidentified by both protein platforms.

Both instrument platforms correctly identified all *A. haemolyticus* samples to the species with the exception of one replicate of each strain classified by the Ultraflextreme at the species with secure genus numeric score values. Both *A. pittii* (formerly genomospecies 3)^24^ strains were correctly identified by the Ultraflextreme with ATCC 17922 given one secure genus, probable species numeric score and 4 probable genus scores. The Microflex system failed twice to provide any ID with the remaining replicates identified correctly with probable genus scores. Both instruments identified *A. pittii* (formerly genomospecies 3)^24^ ATCC 19004.

The Ultraflextreme correctly identified *A. nosocomialis* (formerly genomospecies 13)^24^ ATCC 17903. The Microflex correctly identified it twice with secure genus ID, probable species numerical score values, but misidentified it three times as *A. baumannii*. All five replicates of *A. nosocomialis* (formerly genomospecies 13)^24^ ATCC 700472 were misidentified by Ultraflextreme Biotyper as four different species. The Microflex likewise misidentified this strain as three different species with *A. haemolyticus* as the only common misidentification between the two platforms. Such repeated observation of automated species-level misidentification despite the fact that the Biotyper software gave genus only numerical score values is of concern because of potential misdiagnosis of human pathogens as closely-related but less or non-virulent cousins. Specifically, as an example, *A. baumannii*, a well-characterized pathogen, was misidentified as *A. johnsonii* and *A. tjernbergiae,* two isolates rarely associated with human infection^25^.

### *Listeria* ID

Comparative analyses of two separate strains of five different *Listeria* species (Table 3 and Supplementary Table 3) were conducted using the Biotyper *Listeria*-specific database. The Ultraflextreme platform identified *L. monocytogenes* ATCC 19115 once with a secure genus, probable species numerical score value. The remaining four samples were misidentified as *L. innocua*. Similarly, the Microflex system identified this strain twice to the species, but misidentified it three times as *L. innocua*.

The Ultraflextreme Biotyper identified three replicates of *L. monocytogenes* ATCC 13932. The two remaining samples were incorrectly identified as *L. innocua*. In comparison, the Microflex correctly identified this strain four times, but misidentified it once as *L. innocua*.

Again, automated alphanumeric species-level protein ID outputs were repeatedly observed with only secure genus numerical score values to support such outcomes. This is problematic, especially when it concerns misidentifying a particularly dangerous food-borne pathogen such as *L. monocytogenes* as *L. innocua,* which is only rarely associated with human disease in immunocompromised patients^26^,^27^. This is perplexing given that Bruker has developed a *Listeria*-specific database explicitly for analysis of this genus.

Both platforms identified both *L. grayi* strains to the species level in all instances. However, the Ultraflextreme assigned alphanumeric species ID to *L. grayi* subsp. *grayi* WSLC 6036 and *L. grayi* subsp. *murrayi* WSLC 6037 twice with secure genus, probable species numeric score values, and three times with only probable genus scores. The Microflex system correctly identified all five replicates with secure genus ID, probable species ID numeric score values.

The Ultraflextreme platform identified three replicates of *L. seeligeri* WSLC 40126. It also misidentified this strain once as *L. welshimeri* and failed once to provide any ID. The Microflex Biotyper similarly correctly identified this strain twice but also misidentified it once as *L. monocytogenes* and twice as *L. innocua*.

Both platforms identified *L. seeligeri* WSLC 40127 twice to the species level (the Ultraflextreme once with a secure genus, probable species numeric score value and once with only a probable genus score, the Microflex twice to the species but with only probable genus numeric score values). Both also misidentified this strain twice as *L. monocytogenes*, and once as *L. innocua*.

*L. welshimeri* WSLC 50146 was alphanumerically identified four times to the species, twice with secure genus, probable species scores, and twice with genus only scores. The Ultraflextreme failed to ID the fifth replicate. The Microflex platform identified this strain four times to the species but with only probable genus scores. *L. welshimeri* WSLC 50150 was identified by the Ultraflextreme in all five trials to the species, twice with secure genus, probable species numeric score values and three times with only probable genus values. The Microflex platform identified this strain four times to the species (one secure genus, probable species score value, and three probable genus scores), and misidentified it once as *L. monocytogenes*.

Both instruments correctly identified *L. innocua* ATCC 33090. The Ultraflextreme system misidentified *L. ivanovii* ATCC 19119 once as *L. monocytogenes*, and four times as *L. innocua*. The Microflex platform had similar issues with this strain, misidentifying it four times as *L. monocytogenes*, and once as *L. innocua*.

### Comparison of CeO_2_-catalyzed MOLI MS FA and protein profiling accuracy

A summary of comparative accuracies of CeO_2_-catalyzed MOLI MS results to those observed using both protein profiling platforms is shown in Table 4. It should be noted that for the purposes of a strain-by-strain comparison of FA profiling to protein profiling, the FA spectral database contained fewer entries than the commercial Bruker Biotyper library. It is a possibility that as future work increases the size of the FA database, accuracy could be negatively impacted. However, this is speculative at present and must be borne out by further work. The percentages in Table 4 reflect the number of correct IDs in each category divided by the total number of samples in that category. CeO_2_–catalyzed FA profiling gave 100% correct ID at the genus and species level, while 2% (a single *A. baumannii* AC54) was misidentified as an incorrect strain of the correct species. In contrast, for the three bacterial groups analyzed on two protein profiling instruments, the Biotyper provided 67% (Ultraflextreme and Microflex analysis of *Enterobacteriaceae*) and 96% (Ultraflextreme analysis of *Listeria*) to 100% (Microflex analysis of *Listeria*) correct ID at the genus level. High genus-level accuracy was expected for *Listeria* given the use of the Bruker *Listeria* genus-specific database. Accuracies ranging from 30% (Ultraflextreme analysis of *Enterobacteriaceae*) to 68% (Microflex analysis of *Listeria*) were observed at the species level. Twenty-four percent (Ultraflextreme analysis of *Acinetobacter*) to 33% (Microflex analysis of *Enterobacteriaceae*) were misidentified as an incorrect genus/species and a total Biotyper ID failure rate of 0-18% was observed across the entire study.

**Figure 2:**
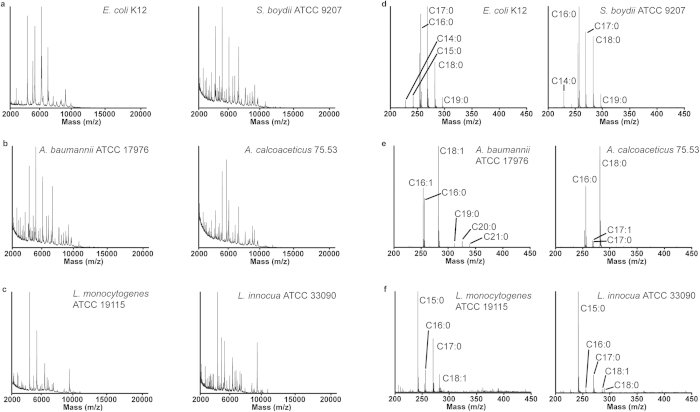
Comparison of protein and CeO_2_-catalyzed fatty acid mass spectral profiling. Representative protein (**a**-**c**) and CeO_2_-catalyzed FA spectra (**d**-**f**) of *E. coli* K12 and *Shigella boydii* ATCC 9207, *A. baumannii* ATCC 17976 and *A. calcoaceticus* 75.53, and *L. monocytogenes* ATCC 19115 and *L. innocua* ATCC 33090, respectively.

Representative FA and protein spectra for bacteria that were misidentified by the Biotyper are shown in Fig. 2. Without knowledge of the proprietary Biotyper software architecture it is uncertain, but it appears that minor protein peaks are not considered and that the major peaks are too similar for a distinction to be made. As examples, comparison of spectra of *E. coli* K12 and *S. boydii* ATCC 9207 (Fig. 2a) showed the same four major protein peaks leading to the misidentification of *S. boydii* as *E. coli*. Despite the fact that *A. calcoaceticus* 75.53 was misidentified as *A. baumannii*, comparison of the protein spectra for these two strains appear visually quite different (Fig. 2b). The largest protein peaks in the spectra of *L. monocytogenes* ATCC 13932 and *L. innocua* ATCC 33090 (Fig. 2c) are nearly identical, with only small differences in intensity. In cases where correct assignments for other species were observed (data not shown), major differences were required in the protein distributions for correct ID.

Although the same major FAs are observed as the molecular weight minus a proton [M-H]^-^, in each of the representative FA spectra shown in Fig. 2d–f, the relative peak intensities, as well as the presence of varying minor FAs, readily allowed for visual and statistical sample differentiation. A specific example is seen in Fig. 2e where *A. baumannii* is visually different than *A. calcoaceticus* because of the appearance of C19:0, C20:0, and C21:0 peaks, which were not observed in the latter.

## Discussion

We address a fundamental question currently surrounding MS-based bacterial ID: which bacterial analyte among a number of possible targets gives the highest possible clinical diagnostic accuracy using modern, commercial MALDI-TOF MS platforms? More specifically, is there a bacterial biomarker with taxonomically useful properties that readily lends itself to rapid MALDI ID without confounding results due to the similarity of that biomolecule across closely related members of clinically important genera? The ideal MS diagnostic system should achieve rapid, accurate, reproducible results with minimal false ID, and do so using a relatively inexpensive, high-throughput, user-friendly assay. This could be achieved by exploiting analytes whose expression and/or composition does not change appreciably in response to environmental conditions such as nutrient availability, temperature, or pH, and that are present in sufficient amounts to allow detection. It is widely acknowledged that expression of bacterial proteins and FAs (two of the most extensively exploited sources of taxonomic biomarkers for bacterial identification) can vary significantly in response to variations in environmental stimuli^28^,^29^,^30^. It is for this reason that precise culturing methods have been developed and adopted for sample preparation for both FA and protein analysis. By adhering to strict culturing practices (e. g. precise media formulation, carefully maintained incubation temperature, and consistent culture duration), such variations are easily minimized and standard operating procedures (SOPs) provided by diagnostic instrument manufacturers have made reproducible sample preparation a matter of routine.

Current protein-based MALDI-TOF MS diagnostic instruments have recently experienced a rapid increase in clinical use with the Bruker Biotyper and bioMérieux VITEK MS platforms. Both these systems draw on the tenants of protein-based bacterial differentiation first realized by Holland *et al.* in the 1990s^31^,^32^. While reasonably rapid and user friendly, we found that the Biotyper suffered from some fundamental drawbacks that limit its accuracy and overall utility. Based on our results and those of others^14^,^15^, it is clear that this system cannot differentiate closely related bacterial species that in many cases express very similar if not identical proteins with similar peak intensities^14^. These problems often result in the reporting of incorrect IDs or failure to provide any reliable ID at all. Most notably, this is borne out by the observation of the misidentification of virulent *A. baumannii* as the closely related, but less problematic, *A. calcoaceticus* (Table 2 and Supplementary Table 2). Perhaps even more concerning was the observation of Biotyper misidentification of *L. monocytogenes* as rarely pathogenic *L. innocua* (Table 3 and Supplementary Table 3), which has the potential for significant negative clinical implications.

Despite the current commercial proliferation (manufacturers quote sales figures of several hundred units per year) of protein-based instruments, early studies pointed towards the potential of FA-based bacterial ID. These investigations demonstrated that bacterial lipid composition successfully correlated to taxonomical trends based on extracted, derivatized FAs followed by gas chromatography (GC)^33^,^34^,^35^,^36^,^37^. Since the 1990s the commercial MIDI platform has been offered for FA methyl ester-based diagnostic ID, but has been limited by the time requirements of the assay^38^.

By coupling the catalytic activity of CeO_2_ with MALDI laser energy, it was observed that bacterial lipids decomposed into taxonomically useful FA constituents. Novel CeO_2_-catalyzed MOLI MS allowed highly accurate ID of species that were routinely misidentified by the Biotyper. Multivariate statistical analysis of the resulting spectra allowed for rapid, highly accurate strain-level ID and differentiation of *Enterobacteriaceae*, *Acinetobacter*, and *Listeria*. Our FA analysis in comparison to the Biotyper provided higher resolution, with 98% accuracy at the strain level and 100% at the species level.

In summary, we have shown by comparison of FA and protein profiling that CeO_2_-catalyzed MOLI MS offers a potentially powerful approach to bacterial ID. We validated method accuracy with sound statistical procedures across a collection of clinically important species that are known to be difficult to differentiate by protein profiling. The primary advantages of this new diagnostic technique are the avoidance of misidentification of closely related species; it has the potential to provide strain-level capabilities, and improve accuracy where current technologies often lack genus- or species-level ID capabilities. Future work will be required to completely evaluate the full capabilities of the technology for bacterial ID and other lipid analyses, construct a comprehensive database, and further develop MOLI MS FA profiling as either a stand-alone technique, or as a means for augmenting existing commercial protein-based systems.

## Methods

### Bacterial strains and growth conditions

*E. coli*, *Salmonella,* and *Shigella* strains were purchased from the American Type Culture Collection (ATCC)(Manassas, VA). *Acinetobacter* strains were obtained from the ATCC and the Felix d’Herelle Reference Center for Bacterial Viruses (University of Laval, Québec, Canada). *Listeria* strains were purchased from the ATCC or were contributed by Dr. Martin Loessner (Institute of Food, Nutrition and Health, Zurich, Switzerland). *Enterobacteriaceae* were cultured for 18 hours in Luria Bertani (LB) broth (Becton Dickinson Difco, Franklin Lakes, NJ) and streaked to isolation on LB agar. *Acinetobacter* and *Listeria* were cultured for 18 hours in brain heart infusion broth (BHI, BD-Difco) and streaked to isolation on BHI agar. All incubation was performed at a constant 37 ^o^C +/−0.2^o^ in strict accordance with Bruker Biotyper SOPs for bacterial cultivation in order to minimize temperature fluctuation-induced changes in FA or protein expression.

### 16S rRNA sequencing and analysis

16S rRNA gene sequencing was used as a simple means to verify the ID of strains in our collection prior to FA and protein analysis. Colonies representing each morphological type were streaked to isolation on BHI or LB agar and incubated for 18 hours at 37 °C under aerobic conditions. Following incubation, single colonies were suspended in 50 μL sterile water, and colony PCR performed using 16S rRNA gene eubacterial oligonucleotide primers 27 F and 1492R (Integrated DNA Technologies, Coralville, IA) to amplify all nine variable regions of the 16 s rRNA gene as previously described^22^. Each 50 μL PCR mixture was composed of 1 μL of colony suspension, 1 μL each of 10 μM forward primer 27 F and reverse primer 1492R, 25 μL DreamTaq 2X master mix (Thermo Scientific), and 22 μL sterile water. Thermal cycling conditions consisted of an initial 3-min denaturation step at 94 °C, 30 cycles of 94 °C for 30 s, 55 °C for 90 s, and 72 °C for 2.5 min, and a final 10-min extension at 72 °C. All reactions were carried out in 0.2 mL reaction tubes using a Techne TC-412 thermocycler (Techne, Burlington, NJ) and PCR products were confirmed by electrophoresis through a 1% agarose gel visualized by ethidium bromide staining. Sequencing reactions were performed by Davis Sequencing (Davis, CA), using an Applied Biosystems 3730 DNA Analyzer (Applied Biosystems, Foster City, CA). Prior to sequencing, all PCR products were treated with ExoSAP-It (Affymetrix, Santa Clara, CA). PCR products were sequenced using the same universal eubacterial 16 S rRNA primers 27 F and 1492R^22^. All 16 S rRNA sequences were compiled and aligned using Geneious 5.5.7 bioinformatics software (Biomatters Ltd, Auckland, New Zealand). All sequences were compared to the sequences in Ribosomal Database Project II (RDP-II)^39^, using the Sequence Match function and by nucleotide BLAST comparison to the NCBI sequence database. Prior to phylogenetic analysis, each sequence was manually edited by examination of its sequencing chromatogram and tested as a possible chimera using Bellerophon^40^. Chimeric sequences and poor-quality sequences were excluded from further analysis. Probable isolate ID was determined as previously described^21^,^22^,^23^.

### Metal oxide sample preparation

#### Lipid extraction

For CeO_2_-catalyzed FA analysis, individual colonies (or a few morphologically identical colonies if small colony size prevented adequate sample preparation from a single colony) of each species were extracted in accordance with Bruker’s SOP for alternative Biotyper whole-cell suspension^20^ with the following modifications: colonies were suspended in 100 μL of a 33/66 v/v% methanol/chloroform (Pharmco-AAPER, Shelbyville KY and Fischer, Pittsburgh PA, respectively) mixture and vortexed for 2 minutes to allow for disruption of the cells as previously described^41^,^42^. Phase separation was achieved by the addition of 100 μL of phosphate buffer saline (PBS) pH 7.4 to facilitate lipid extraction. Mixtures were then vortexed for another 60 seconds and centrifuged briefly prior to MALDI sample plate spotting.

#### Mass spectrometry

Samples were prepared for mass spectrometry as previously described^11^,^12^. Briefly, 100 mg of CeO_2_ (Nano-Active Inc. Manhattan KS) was added to one mL of *n*-hexane. One μL was then removed from the resulting slurry and spotted on a stainless steel MALDI sample plate. Two μL lipid extract aliquots were spotted directly onto activated CeO_2_ spots. Negative controls were run on SBA-15 (Sigma-Aldrich, St. Louis, MO) to ensure FA spectra were the result of CeO_2_ catalysis and not from thermal desorption of lipids. All data was obtained in negative-ion mode as previously described^12^.

#### FA data analysis

Twenty-three FA peaks (Shown in Supplementary Table 7) from the mass spectra data were selected, centroided, assigned nominal masses, and compiled into a spreadsheet using software written in-house. The spreadsheet was imported into the R Statistics Software program (Ver. 3.0.2, R Foundation, Vienna, Austria) for PCA and leave-one-out CV. The *prcomp* function was used to calculate PCA scores; *prcomp* mean-centers the data, but no auto-scaling was used. Scores from PCA analysis were plotted using the *plot* function. CV was done using the *lda* function from the Modern Applied Statistics with S (MASS) package^43^ and setting the “CV” flag as “True.” Results from CV are reported as percentages of correct assignments divided by total measurements.

#### MALDI Biotyper sample preparation

Five replicates of single morphologically distinct colonies of each strain were collected from agar plates and each applied to separate wells on polished stainless steel MALDI target plates (Bruker Daltonic, Billerica, MA). Samples were then overlaid with 1 μL of α-cyano-4-hydroxycinnamic acid matrix (HCCA) (Bruker) according to the manufacturer’s instructions. MALDI plates were then placed in sterile, covered petri dishes and allowed to air dry at room temperature.

#### MALDI protein analysis

Protein analysis was conducted using Bruker Ultraflextreme and Microflex MALDI-TOF mass spectrometers configured to run Bruker Biotyper Real Time Classification (RTC) (v3.0) software according to the manufacturer’s instructions. The Ultraflextreme was detuned to match Microflex settings. Specifically, the ion source was fixed in positive-ion mode, sensitivity decreased from 150:1 to 50:1, and resolution decreased from 25,000 to 1000. Detector voltage was variably reduced to lower signal response and the digitizer sampling rate was lowered from 2 GS/s to 0.5 GS/s in order to match Microflex specifications. Five individual replicates of each strain were independently analyzed by automated laser rastering. The instrument was calibrated using the Bruker Biotyper Bacterial Test Standard (BTS) according to the manufacturer’s instructions. Bacterial ID results were automatically generated as a pdf report for each of three groups of bacterial strains (*Enterobacteriaceae*, *Acinetobacter*, and *Listeria*). *Enterobacteriaceae* and *Acinetobacter* were identified using the built-in Bruker Biotyper IVD database, while *Listeria* were analyzed using the Bruker *Listeria*-specific Biotyper database. PCA was conducted using Biotyper 3.0 analysis tools. All samples were subjected to a second round of Biotyper analysis using a pre-analysis sample extraction as recommended by Bruker and as previously described^20^. Briefly, single colonies (or a few morphologically identical colonies if small colony size prevented adequate MALDI-TOF MS sample preparation from a single colony) were suspended in 30 μL of 70% ethanol and centrifuged. Pellets were then resuspended in an equal volume of 70% formic acid and vortexed followed by addition of 30 μL of 100% acetonitrile. Resulting suspensions were centrifuged and supernatants collected for analysis.

## Additional Information

**How to cite this article**: Cox, C. R. *et al.* Strain-level bacterial identification by CeO_2_-catalyzed MALDI-TOF MS fatty acid analysis and comparison to commercial protein-based methods. *Sci. Rep.* **5**, 10470; doi: 10.1038/srep10470 (2015).

## Supplementary Material

Supplementary Information

## Figures and Tables

**Table 1:**
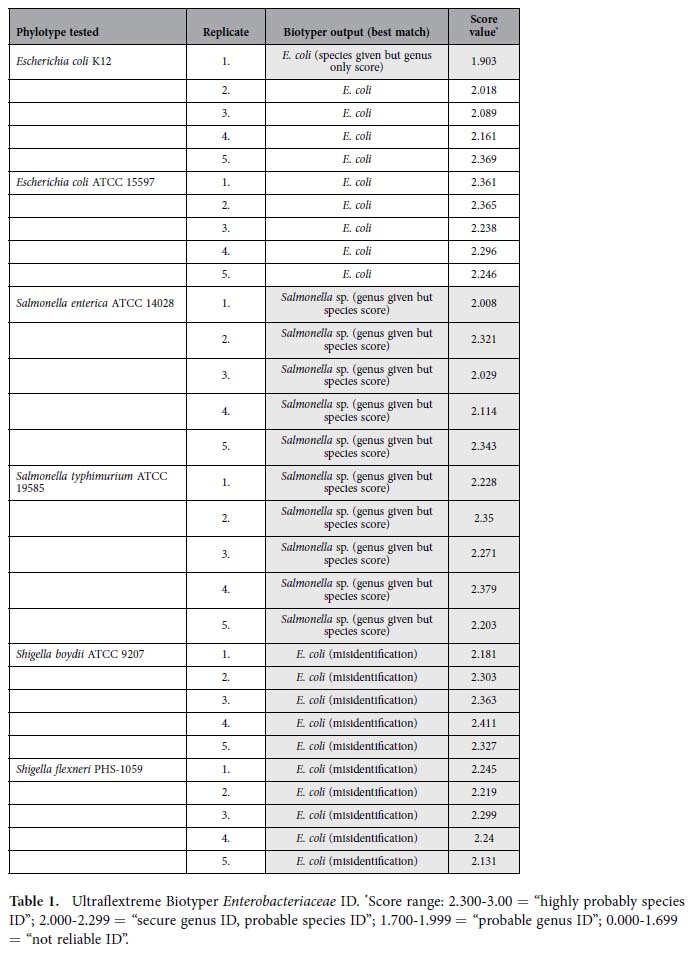
Ultraflextreme Biotyper *Enterobacteriaceae* ID.

**Table 2:**
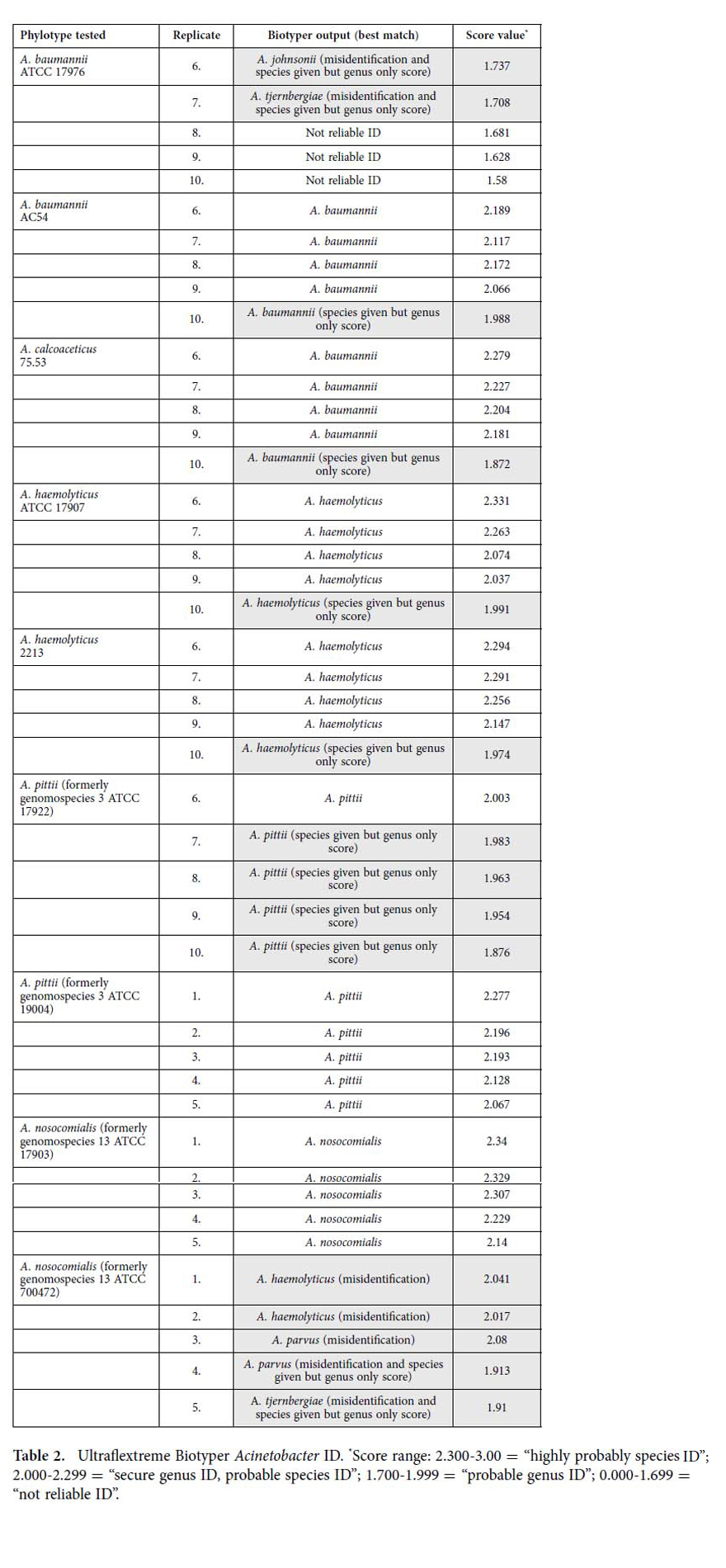
Ultraflextreme Biotyper *Acinetobacter* ID.

**Table 3:**
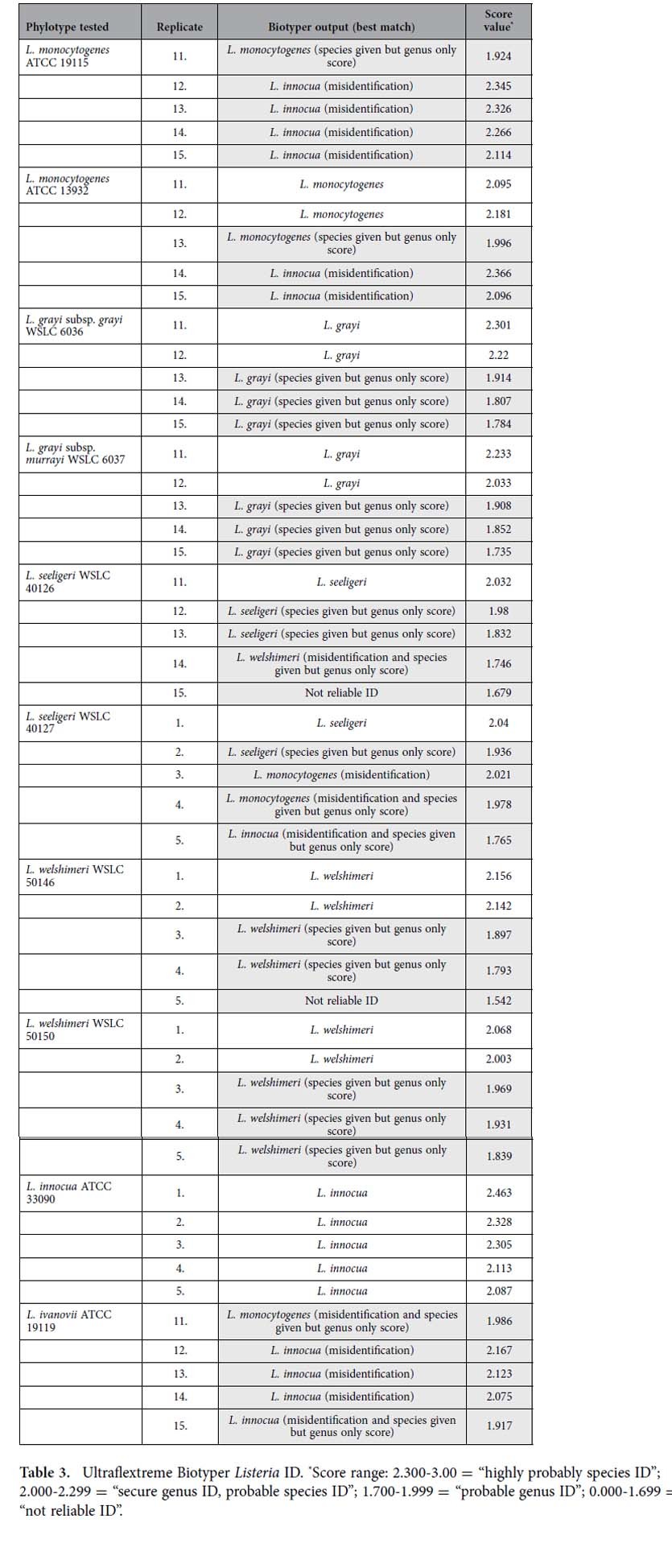
Ultraflextreme Biotyper *Listeria* ID.

**Table 4:** Percent accuracy of Biotyper bacterial ID and CeO_2_-catalyzed MOLI fatty acid analysis.

	CeO_2_-catalyzed MOLI		Ultraflextreme		Microflex	
*Enterobacteriaceae*	Strain	100% (30/30)	Strain	-	Strain	-
	Species	100% (30/30)	Species	30% (9/30)	Species	33% (10/30)
	Genus	100% (30/30)	Genus	67% (20/30)	Genus	67% (20/30)
	Mis-ID	0%	Mis-ID	33% (10/30)	Mis-ID	33% (10/30)
	Failure to ID	0%	Failure to ID	0%	Failure to ID	0%
*Acinetobacter*	Strain	98% (44/45)	Strain	-	Strain	-
	Species	100% (45/45)	Species	64% (29/45)	Species	44% (20/45)
	Genus	100% (45/45)	Genus	84% (38/45)	Genus	82% (37/45)
	Mis-ID	2% (1/45)	Mis-ID	24% (11/45)	Mis-ID	27% (12/45)
	Failure to ID	0%	Failure to ID	7% (3/45)	Failure to ID	18% (8/45)
*Listeria*	Strain	100% (50/50)	Strain	-	Strain	-
	Species	100% (50/50)	Species	66% (33/50)	Species	68% (34/50)
	Genus	100% (50/50)	Genus	96% (48/50)	Genus	100% (50/50)
	Mis-ID	0%	Mis-ID	30% (15/50)	Mis-ID	32% (16/50)
	Failure to ID	0%	Failure to ID	4% (2/50)	Failure to ID	0%
