# Supplementary material for: Strain-level bacterial identification by CeO2-catalyzed MALDI-TOF MS fatty acid analysis and comparison to commercial protein-based methods
Source: Sci Rep. 2015 Jul 20;5:10470. doi: 10.1038/srep10470 (PMC4507139; doi:10.1038/srep10470)
Supplement: Supplementary Information [file srep10470-s1.pdf]

Strain-level bacterial identification by CeO_2-catalyzed MALDI-TOF MS fatty acid analysis and comparison to commercial protein-based methods.

Cox, C.R.*, Jensen, K. R., Saichek, N.R. and Voorhees, K.J.*

Department of Chemistry and Geochemistry, Colorado School of Mines, Golden, CO 80401.

*Corresponding authors.

kvoorhee@mines.edu

crcox@mines.edu

SUPPLEMENTARY DATA

Supplementary Table 1. Microflex Biotyper *Enterobacteriaceae* ID

Phylotype tested	Replicate	Biotyper output (best match)	Score value*
Escherichia coli K12	1.	*E. coli*	1.903
	2.	*E. coli*	2.018
	3.	*E. coli*	2.089
	4.	*E. coli*	2.161
	5.	*E. coli*	2.369
Escherichia coli ATCC 15597	1.	*E. coli*	2.361
	2.	*E. coli*	2.365
	3.	*E. coli*	2.238
	4.	*E. coli*	2.296
	5.	*E. coli*	2.246
Salmonella enterica ATCC 14028	1.	*Salmonella* sp. (genus given but species score)	2.008
	2.	*Salmonella* sp. (genus given but species score)	2.321
	3.	*Salmonella* sp. (genus given but species score)	2.029
	4.	*Salmonella* sp. (genus given but species score)	2.114
	5.	*Salmonella* sp. (genus given but species score)	2.343
Salmonella typhimurium ATCC 19585	1.	*Salmonella* sp. (genus given but species score)	2.228
	2.	*Salmonella* sp. (genus given but species score)	2.35
	3.	*Salmonella* sp. (genus given but species score)	2.271
	4.	*Salmonella* sp. (genus given but species score)	2.379
	5.	*Salmonella* sp. (genus given but species score)	2.203
Shigella boydii ATCC 9207	1.	*E. coli* (misidentification)	2.181
	2.	*E. coli* (misidentification)	2.303
	3.	*E. coli* (misidentification)	2.363
	4.	*E. coli* (misidentification)	2.411
	5.	*E. coli* (misidentification)	2.327
Shigella flexneri PHS-1059	1.	*E. coli* (misidentification)	2.245
	2.	*E. coli* (misidentification)	2.219
	3.	*E. coli* (misidentification)	2.299

	4.	*E. coli* (misidentification)	2.24
	5.	*E. coli* (misidentification)	2.131

*Score range: 2.300-3.00 = “highly probably species ID”; 2.000-2.299 = “secure genus ID, probable species ID”; 1.700-1.999 = “probable genus ID”; 0.000-1.699 = “not reliable ID”.

Supplementary Table 2. Microflex Biotyper *Acinetobacter* ID

Phylotype tested	Replicate	Biotyper output (best match)	Score value*
A. baumannii ATCC 17976	6.	Not reliable ID	1.67
	7.	Not reliable ID	1.639
	8.	Not reliable ID	1.632
	9.	Not reliable ID	1.56
	10.	Not reliable ID	1.535
A. baumannii AC54	6.	*A. baumannii* (species given but genus only score)	1.997
	7.	*A. baumannii* (species given but genus only score)	1.963
	8.	*A. baumannii* (species given but genus only score)	1.943
	9.	*A. baumannii* (species given but genus only score)	1.936
	10.	*A. baumannii* (species given but genus only score)	1.904
A. calcoaceticus 75.53	6.	*A. baumannii* (misidentification)	2.124
	7.	*A. baumannii* (misidentification)	2.108
	8.	*A. baumannii* (misidentification)	2.086
	9.	*A. baumannii* (misidentification and species given but genus only score)	1.989
	10.	*A. nosocomialis* (misidentification and species given but genus only score)	1.96
A. haemolyticus ATCC 17907	6.	*A. haemolyticus*	2.284
	7.	*A. haemolyticus*	2.225
	8.	*A. haemolyticus*	2.209
	9.	*A. haemolyticus*	2.195
	10.	*A. haemolyticus*	2.113
A. haemolyticus 2213	6.	*A. haemolyticus*	2.288
	7.	*A. haemolyticus*	2.274
	8.	*A. haemolyticus*	2.258
	9.	*A. haemolyticus*	2.255
	10.	*A. haemolyticus*	2.224
A. pittii (formerly genomospecies 3 ATCC 17922)	6.	*A. pittii* (species given but genus only score)	1.889
	7.	*A. pittii* (species given but genus only score)	1.833
	8.	*A. pittii* (species given but genus only score)	1.778

	9.	Not reliable ID	1.479
	10.	Not reliable ID	1.276
A. pittii (formerly genomospecies 3 ATCC 19004)	1.	*A. pittii*	2.289
	2.	*A. pittii*	2.243
	3.	*A. pittii*	2.21
	4.	*A. pittii*	2.124
	5.	*A. pittii*	2.046
A. nosocomialis (formerly genomospecies 13 ATCC 17903)	1.	*A. nosocomialis*	2.242
	2.	*A. nosocomialis*	2.144
	3.	*A. baumannii* (misidentification)	2.181
	4.	*A. baumannii* (misidentification)	2.128
	5.	*A. baumannii* (misidentification)	2.116
A. nosocomialis (formerly genomospecies 13 ATCC 700472)	1.	*A. haemolyticus* (misidentification and species given but genus only score)	1.798
	2.	*A. junii* (misidentification and species given but genus only score)	1.876
	3.	*A. junii* (misidentification and species given but genus only score)	1.85
	4.	*A. junii* (misidentification and species given but genus only score)	1.774
	5.	No reliable ID	1.566

*Score range: 2.300-3.00 = “highly probably species ID”; 2.000-2.299 = “secure genus ID, probable species ID”; 1.700-1.999 = “probable genus ID”; 0.000-1.699 = “not reliable ID”.

Table 3. Microflex Biotyper *Listeria* ID

Phylotype tested	Replicate	Biotyper output (best match)	Score value*
L. monocytogenes ATCC 19115	11.	*L. monocytogenes*	2.106
	12.	*L. monocytogenes* (species given, but genus only score)	1.998
	13.	*L. innocua* (misidentification and species given but genus only score)	1.998
	14.	*L. innocua* (misidentification and species given but genus only score)	1.914
	15.	*L. innocua* (misidentification and species given but genus only score)	1.88
L. monocytogenes ATCC 13932	11.	*L. monocytogenes*	2.067
	12.	*L. monocytogenes* (species given but genus only score)	1.97
	13.	*L. monocytogenes* (species given but genus only score)	1.944
	14.	*L. monocytogenes* (species given but genus only score)	1.817
	15.	*L. innocua* (misidentification and species given but genus only score)	1.804
L. grayi subsp. *grayi* WSLC 6036	11.	*L. grayi*	2.158
	12.	*L. grayi*	2.089
	13.	*L. grayi*	2.061
	14.	*L. grayi*	2.057
	15.	*L. grayi*	2.056
L. grayi subsp. *murrayi* WSLC 6037	11.	*L. grayi*	2.117
	12.	*L. grayi*	2.116
	13.	*L. grayi*	2.104
	14.	*L. grayi*	2.067
	15.	*L. grayi*	2.051
L. seeligeri WSLC 40126	11.	*L. seeligeri* (species given but genus only score)	1.963
	12.	*L. seeligeri* (species given but genus only score)	1.867
	13.	*L. monocytogenes* (misidentification)	2.02
	14.	*L. innocua* (misidentification)	2.167
	15.	*L. innocua* (misidentification and species given but genus only score)	1.906

L. seeligeri<br>WSLC 40127	1.	*L. seeligeri*<br>(species given but genus only score)	1.9
	2.	*L. seeligeri*<br>(species given but genus only score)	1.891
	3.	*L. monocytogenes*<br>(misidentification)	2.021
	4.	*L. monocytogenes*<br>(misidentification and species given but genus only score)	1.888
	5.	*L. innocua*<br>(misidentification and species given but genus only score)	1.944
L. welshimeri<br>WSLC 50146	1.	*L. welshimeri*<br>(species given but genus only score)	1.937
	2.	*L. welshimeri*<br>(species given but genus only score)	1.871
	3.	*L. welshimeri*<br>(species given but genus only score)	1.832
	4.	*L. welshimeri*<br>(species given but genus only score)	1.769
	5.	*L. welshimeri*<br>(species given but genus only score)	1.737
L. welshimeri<br>WSLC 50150	1.	*L. welshimeri*	2.03
	2.	*L. welshimeri*<br>(species given but genus only score)	1.955
	3.	*L. welshimeri*<br>(species given but genus only score)	1.922
	4.	*L. welshimeri*<br>(species given but genus only score)	1.849
	5.	*L. monocytogenes*<br>(misidentification)	1.839
L. innocua<br>ATCC 33090	1.	*L. innocua*	2.284
	2.	*L. innocua*	2.249
	3.	*L. innocua*	2.249
	4.	*L. innocua*	2.244
	5.	*L. innocua*	2.114
L. ivanovii<br>ATCC 19119	11.	*L. monocytogenes*<br>(misidentification)	2.169
	12.	*L. monocytogenes*<br>(misidentification)	2.042
	13.	*L. monocytogenes*<br>(misidentification and species given but genus only score)	1.988
	14.	*L. monocytogenes*<br>(misidentification and species given but genus only score)	1.832
	15.	*L. innocua*<br>(misidentification and species given but genus only score)	1.988

*Score range: 2.300-3.00 = "highly probably species ID"; 2.000-2.299 = "secure genus ID, probable species ID"; 1.700-1.999 = "probable genus ID"; 0.000-1.699 = "not reliable ID".

Supplementary Table 4 Leave-one-out cross validation of CeO_2-catalyzed *Enterobacteriaceae* classification

**Strain tested**	**Probability of strain queried**					
	E. coli K12	*E. coli* ATCC 15597	*Salmonella enterica* ATCC 14028	*Salmonella typhimurium* ATCC 19585	*Shigella boydii* ATCC 9207	*Shigella flexneri* PHS-1059
E. coli K12	1	0	0	0	0	0
"	1	0	0	0	0	0
"	1	0	0	0	0	0
"	1	0	0	0	0	0
"	1	0	0	0	0	0
E. coli ATCC 15597	0	1	1.07E-250	0	0	0
"	0	1	1.41E-238	0	0	0
"	0	1	4.39E-245	0	0	0
"	0	1	1.78E-237	0	0	0
"	0	1	3.74E-257	0	0	0
Salmonella enterica ATCC 14028	4.96E-237	0	1	0	2.95E-157	0
"	3.09E-274	0	1	0	1.51E-163	0
"	0.00E-01	0	1	0	1.65E-166	0
"	1.57E-264	0	1	0	3.60E-225	0
"	4.67E-264	0	1	0	9.75E-163	0
Salmonella typhimurium ATCC 19585	0	0		1	0	0
"	0	0	0	1	0	0
"	0	0	0	1	0	0
"	0	0	0	1	0	0
"	0	0	0	1	0	0
Shigella boydii ATCC 9207	0	0	6.51E-201	0	1	0
"	0	0	2.06E-193	0	1	0
"	0	0	1.09E-169	0	1	0

“	0	0	9.47E-176	0	1	0
“	0	0	1.25E-187	0	1	0
Shigella flexneri PHS-1059	0	0	0	0	0	1
“	0	0	0	0	0	1
“	0	0	0	0	0	1
“	0	0	0	0	0	1
“	0	0	0	0	0	1

Supplementary Table 5 Leave-one-out cross validation of CeO_2-catalyzed *Acinetobacter* classification

Strain tested	Probability of strain queried								
	A. baumannii ATCC 17976	*A. baumannii* AC54	*A. calcoaceticus* 75.53	*A. haemolyticus* ATCC 17907	*A. haemolyticus* 2213	*A. pittii* ATCC 17922	*A. pittii* ATCC 19004	*A. nosocomialis* ATCC 17903	*A. nosocomialis* ATCC 700472
A. baumannii ATCC 17976	0.999	0.0001	0	0	0	0	0	0	4.31E-115
"	1	4.49E-19	0	0	0	0	0	0	1.16E-124
"	1	2.93E-21	0	0	0	0	0	0	3.10E-154
"	1	2.51E-36	0	0	0	0	0	0	1.53E-162
"	1.90E-07	0.9999	0	0	0	0	0	0	5.88E-119
A. baumannii AC54	1.28E-14	1	0	0	0	0	0	0	2.73E-104
"	5.04E-14	1	0	0	0	0	0	0	1.85E-103
"	2.10E-07	0.999	0	0	0	0	0	0	1.44E-131
"	9.22E-11	1	0	0	0	0	0	0	3.15E-103
"	1.08E-09	0.999	0	0	0	0	0	0	1.94E-160
A. calcoaceticus 75.53	0	0	1	0	2.33E-304	0	0	0	0
"	0	0	1	0	2.81E-307	0	0	0	0
"	0	0	1	0	0	0	0	0	0
"	0	0	1	0	0	0	0	0	0
"	0	0	1	0	0	0	0	0	0
A. haemolyticus ATCC 17907	0	0	0	1	1.09E-140	4.45E-92	1.29E-70	0	0
"	0	0	0	1	4.73E-123	5.41E-87	4.62E-67	0	0
"	0	0	0	1	2.12E-136	1.61E-85	4.84E-62	0	0
"	0	0	0	1	3.36E-121	2.17E-93	3.65E-60	0	0
"	0	0	0	1	1.45E-139	2.12E-96	5.71E-68	0	0
A. haemolyticus 2213	0	0	8.10E-305	1.23E-145	1	3.73E-208	3.06E-142	0	0
"	0	0	0	8.41E-117	1	3.78E-171	8.61E-109	0	0

“	0	0	6.21E-320	1.30E-122	1	2.91E-176	2.37E-115	0	0
“	0	0	4.62E-317	5.95E-126	1	1.88E-184	1.19E-121	0	0
A. pittii ATCC 17922	0	0	0	4.14E-99	6.64E-210	1	6.83E-39	0	0
“	0	0	0	2.57E-93	5.46E-174	1	5.32E-20	0	0
“	0	0	0	1.62E-88	4.86E-177	1	2.04E-24	0	0
“	0	0	0	2.38E-82	8.66E-192	1	5.92E-31	0	0
“	0	0	0	3.90E-94	1.02E-181	1	5.08E-31	0	0
A. pittii ATCC 19004	0	0	0	1.02E-65	5.23E-113	2.69E-25	1	0	0
“	0	0	0	3.89E-81	1.27E-135	6.18E-24	1	0	0
“	0	0	0	9.82E-57	1.66E-122	9.64E-41	1	0	0
“	0	0	0	1.02E-63	3.71E-113	9.81E-35	1	0	0
“	0	0	0	7.39E-67	6.87E-131	9.53E-23	1	0	0
A. nosocomialis ATCC 17903	0	0	0	0	0	0	0	1	0
“	0	0	0	0	0	0	0	1	0
“	0	0	0	0	0	0	0	1	0
“	0	0	0	0	0	0	0	1	0
“	0	0	0	0	0	0	0	1	0
A. nosocomialis ATCC 700472	4.50E-123	2.15E-110	0	0	0	0	0	0	1
“	1.75E-126	9.96E-111	0	0	0	0	0	0	1
“	5.77E-136	3.90E-132	0	0	0	0	0	0	1
“	3.25E-123	5.90E-106	0	0	0	0	0	0	1
“	1.09E-128	2.27E-108	0	0	0	0	0	0	1

Supplementary Table 6 Leave-one-out cross validation of CeO_2-catalyzed *Listeria* classification

Strain tested	Probability of strain queried								
	L. monocytogenes ATCC 19115	*L. monocytogenes* ATCC 13932	*L. grayi* subsp. grayi WSLC 6036	*L. grayi* subsp. murrayi WSLC 6037	*L. seeligeri* WSLC 40127	*L. seeligeri* WSLC 40126	*L. welshimeri* WSLC 50146	*L. welshimeri* WSLC 50150	*L. innocua* ATCC 33090	*L. ivanovii* ATCC 19119
L. monocytogenes ATCC 19115	1	2.77E-40	4.15E-304	3.25E-43	2.14E-54	1.01E-103	7.89E-40	1.55E-38	6.90E-252	1.96E-48
"	1	7.47E-35	0.00E+00	2.48E-36	6.11E-42	1.38E-93	2.16E-32	6.28E-43	3.13E-247	3.04E-40
"	1	1.10E-32	2.16E-288	7.40E-35	7.76E-47	7.44E-92	1.68E-37	4.23E-44	1.10E-247	8.71E-49
"	1	3.40E-36	1.20E-284	3.91E-35	8.69E-48	2.01E-87	4.07E-34	9.89E-34	1.51E-235	6.15E-46
"	1	1.13E-29	1.66E-293	8.27E-32	1.66E-41	2.51E-86	1.75E-31	3.07E-44	8.25E-234	1.90E-40
L. monocytogenes ATCC 13932	1.56E-33	0.999	1.13E-300	1.69E-05	7.62E-57	1.11E-85	1.42E-58	3.11E-118	1.43E-181	5.74E-81
"	1.47E-29	0.995	5.22E-301	0.004	7.85E-38	1.03E-70	1.08E-40	4.74E-109	3.04E-168	9.80E-59
"	6.71E-32	0.999	0	7.38E-09	3.61E-44	7.99E-81	2.33E-46	9.82E-115	1.37E-168	1.04E-61
"	1.50E-37	0.985	8.18E-301	0.015	3.75E-49	2.81E-75	7.14E-50	1.71E-118	2.07E-159	8.56E-72
"	2.81E-39	0.999	0	7.96E-07	8.12E-44	7.46E-78	1.96E-48	1.04E-134	3.44E-166	1.28E-65
L. grayi subsp. grayi WSLC 6036	0	0	1	0	0	5.48E-179	0	5.24E-273	0	0
"	1.59E-277	0	1	2.91E-283	0	7.60E-197	6.34E-310	3.57E-215	0	0
"	7.25E-297	2.2E-316	1	2.43E-269	0	2.06E-164	1.12E-304	1.50E-224	0	0
"	0	0	1	0	0	2.21E-299	0	0	0	0
"	2.65E-287	0	1	5.61E-276	0	1.25E-169	8.26E-312	5.56E-220	0	0
L. grayi subsp. murrayi WSLC 6037	4.30E-36	1.82E-06	4.34E-261	0.999	3.56E-37	2.53E-60	2.83E-35	2.77E-106	4.39E-141	1.70E-59
"	4.71E-30	1.03E-05	3.58E-262	0.999	1.65E-30	3.16E-58	4.89E-29	3.33E-102	4.53E-147	3.06E-51
"	8.35E-40	3.30E-05	3.49E-296	0.999	9.48E-32	4.44E-69	4.75E-33	3.09E-126	1.31E-	2.90E-53

									145	
"	1.22E-43	1.05E-05	1.37E-261	0.999	9.72E-43	5.87E-62	2.60E-42	9.12E-117	6.60E-139	2.06E-70
"	1.00E-34	7.62E-05	3.42E-283	0.999	1.05E-33	6.66E-69	2.76E-34	4.11E-111	6.89E-154	7.47E-55
L. seeligeri WSLC 40127	2.64E-50	1.82E-50	0	1.22E-38	0.999	4.60E-68	1.18E-07	3.39E-112	1.29E-193	6.10E-09
"	5.29E-50	3.70E-72	0	7.17E-55	0.913	7.63E-74	0.010	3.81E-102	2.88E-205	0.076
"	4.61E-47	1.41E-42	0	2.96E-32	0.999	2.87E-69	5.71E-09	1.20E-116	8.82E-194	1.50E-10
"	9.15E-40	2.51E-39	0	1.22E-28	0.999	1.31E-63	4.53E-05	2.57E-101	1.60E-188	7.01E-10
"	6.72E-45	2.25E-39	0	1.27E-29	1	3.40E-69	7.17E-11	7.72E-116	1.33E-199	1.89E-13
L. seeligeri WSLC 40126	1.24E-87	6.62E-73	7.93E-171	1.97E-56	8.22E-62	1	1.40E-48	2.81E-97	1.35E-87	2.29E-89
"	4.43E-87	5.47E-78	1.03E-192	8.24E-64	3.12E-73	1	1.72E-51	1.09E-84	1.45E-79	8.05E-98
"	8.35E-98	2.71E-89	5.68E-176	5.79E-70	3.19E-58	1	4.07E-48	1.50E-104	8.73E-128	3.34E-87
"	1.51E-85	9.47E-69	6.08E-168	7.91E-53	2.99E-66	1	1.55E-48	3.43E-81	2.08E-74	2.12E-91
"	1.04E-92	2.80E-71	1.45E-174	1.08E-67	1.12E-87	1	9.97E-86	4.52E-117	1.70E-125	5.20E-129
L. welshimeri WSLC 50146	8.83E-34	2.04E-50	6.94E-300	6.68E-32	7.97E-08	4.95E-58	0.99	6.07E-74	4.21E-166	1.34E-10
"	5.35E-33	2.35E-61	0	5.85E-46	2.10E-05	1.13E-68	0.999	2.22E-73	4.17E-220	1.23E-05
"	3.53E-33	1.69E-40	1.19E-299	1.14E-26	1.78E-07	2.04E-48	0.999	3.62E-72	1.58E-154	4.67E-13
"	9.69E-35	1.42E-43	0	2.50E-31	0.0002	7.51E-60	0.999	3.46E-82	1.14E-165	3.92E-07
"	5.19E-37	6.73E-51	8.49E-307	1.51E-39	2.41E-09	1.33E-45	0.999	6.29E-69	7.07E-153	1.41E-11
L. welshimeri WSLC 50150	1.64E-37	6.92E-121	6.38E-248	5.67E-114	7.84E-104	5.88E-98	6.06E-69	1	1.28E-277	9.26E-97
"	7.64E-68	2.08E-170	1.07E-228	5.97E-164	3.31E-167	7.60E-122	5.68E-123	1	3.29E-312	2.30E-157
"	2.20E-37	2.62E-109	1.03E-219	8.75E-102	1.77E-104	3.66E-88	2.13E-67	1	8.09E-244	2.04E-94

“	7.28E-31	2.03E-110	6.55E-222	2.52E-102	8.71E-100	1.91E-86	5.76E-65	1	7.81E-248	9.75E-89
“	3.77E-34	3.43E-117	2.87E-248	2.16E-106	1.17E-100	1.09E-99	5.09E-68	1	5.26E-249	5.92E-84
L. innocua ATCC 33090	7.87E-229	1.04E-153	6.32E-305	4.22E-131	1.86E-185	2.35E-81	7.81E-152	1.24E-243	1	1.81E-213
“	2.60E-286	2.72E-217	0	5.21E-184	1.78E-224	4.61E-102	1.08E-187	1.19E-280	1	1.29E-254
“	0	1.58E-224	0	3.72E-199	1.18E-280	7.70E-130	4.13E-235	0	1	2.05E-316
“	5.27E-248	1.13E-167	0	2.82E-146	9.97E-198	7.31E-95	3.98E-167	5.04E-275	1	9.42E-227
“	2.21E-273	1.66E-175	4.66E-287	3.94E-133	2.23E-180	6.33E-65	7.85E-143	2.67E-293	1	2.13E-218
L. ivanovii ATCC 19119	9.91E-36	2.02E-59	0	3.21E-50	7.19E-08	7.15E-98	1.16E-07	1.37E-89	1.00E-236	0.999
“	3.34E-35	2.45E-57	0	2.02E-47	0.0001	3.50E-94	6.70E-06	3.48E-91	1.76E-228	0.999
“	7.56E-46	4.79E-72	0	4.82E-63	1.83E-09	3.25E-83	6.79E-07	1.76E-86	1.83E-209	0.999
“	3.58E-59	2.35E-74	0	8.12E-65	1.92E-09	8.90E-110	3.54E-14	2.01E-123	1.24E-229	0.999
“	6.71E-60	8.41E-79	0	5.57E-66	9.71E-11	1.30E-106	6.68E-11	1.22E-116	6.34E-220	1

Supplementary Table 6 Leave-one-out cross validation of CeO_2-catalyzed *Listeria* classification

Strain tested	Probability of strain queried									
	L. monocytogenes ATCC 19115	*L. monocytogenes* ATCC 13932	*L. grayi* subsp. grayi WSLC 6036	*L. grayi* subsp. murrayi WSLC 6037	*L. seeligeri* WSLC 40127	*L. seeligeri* WSLC 40126	*L. welshimeri* WSLC 50146	*L. welshimeri* WSLC 50150	*L. innocua* ATCC 33090	*L. ivanovii* ATCC 19119
L. monocytogenes ATCC 19115	1	2.77E-40	4.15E-304	3.25E-43	2.14E-54	1.01E-103	7.89E-40	1.55E-38	6.90E-252	1.96E-48
"	1	7.47E-35	0.00E+00	2.48E-36	6.11E-42	1.38E-93	2.16E-32	6.28E-43	3.13E-247	3.04E-40
"	1	1.10E-32	2.16E-288	7.40E-35	7.76E-47	7.44E-92	1.68E-37	4.23E-44	1.10E-247	8.71E-49
"	1	3.40E-36	1.20E-284	3.91E-35	8.69E-48	2.01E-87	4.07E-34	9.89E-34	1.51E-235	6.15E-46
"	1	1.13E-29	1.66E-293	8.27E-32	1.66E-41	2.51E-86	1.75E-31	3.07E-44	8.25E-234	1.90E-40
L. monocytogenes ATCC 13932	1.56E-33	0.999	1.13E-300	1.69E-05	7.62E-57	1.11E-85	1.42E-58	3.11E-118	1.43E-181	5.74E-81
"	1.47E-29	0.995	5.22E-301	0.004	7.85E-38	1.03E-70	1.08E-40	4.74E-109	3.04E-168	9.80E-59
"	6.71E-32	0.999	0	7.38E-09	3.61E-44	7.99E-81	2.33E-46	9.82E-115	1.37E-168	1.04E-61
"	1.50E-37	0.985	8.18E-301	0.015	3.75E-49	2.81E-75	7.14E-50	1.71E-118	2.07E-159	8.56E-72
"	2.81E-39	0.999	0	7.96E-07	8.12E-44	7.46E-78	1.96E-48	1.04E-134	3.44E-166	1.28E-65
L. grayi subsp. grayi WSLC 6036	0	0	1	0	0	5.48E-179	0	5.24E-273	0	0
"	1.59E-277	0	1	2.91E-283	0	7.60E-197	6.34E-310	3.57E-215	0	0
"	7.25E-297	2.2E-316	1	2.43E-269	0	2.06E-164	1.12E-304	1.50E-224	0	0
"	0	0	1	0	0	2.21E-299	0	0	0	0
"	2.65E-287	0	1	5.61E-276	0	1.25E-169	8.26E-312	5.56E-220	0	0

L. grayi subsp. murrayi WSLC 6037	4.30E-36	1.82E-06	4.34E-261	0.999	3.56E-37	2.53E-60	2.83E-35	2.77E-106	4.39E-141	1.70E-59
"	4.71E-30	1.03E-05	3.58E-262	0.999	1.65E-30	3.16E-58	4.89E-29	3.33E-102	4.53E-147	3.06E-51
"	8.35E-40	3.30E-05	3.49E-296	0.999	9.48E-32	4.44E-69	4.75E-33	3.09E-126	1.31E-145	2.90E-53
"	1.22E-43	1.05E-05	1.37E-261	0.999	9.72E-43	5.87E-62	2.60E-42	9.12E-117	6.60E-139	2.06E-70
"	1.00E-34	7.62E-05	3.42E-283	0.999	1.05E-33	6.66E-69	2.76E-34	4.11E-111	6.89E-154	7.47E-55
L. seeligeri WSLC 40127	2.64E-50	1.82E-50	0	1.22E-38	0.999	4.60E-68	1.18E-07	3.39E-112	1.29E-193	6.10E-09
"	5.29E-50	3.70E-72	0	7.17E-55	0.913	7.63E-74	0.010	3.81E-102	2.88E-205	0.076
"	4.61E-47	1.41E-42	0	2.96E-32	0.999	2.87E-69	5.71E-09	1.20E-116	8.82E-194	1.50E-10
"	9.15E-40	2.51E-39	0	1.22E-28	0.999	1.31E-63	4.53E-05	2.57E-101	1.60E-188	7.01E-10
"	6.72E-45	2.25E-39	0	1.27E-29	1	3.40E-69	7.17E-11	7.72E-116	1.33E-199	1.89E-13
L. seeligeri WSLC 40126	1.24E-87	6.62E-73	7.93E-171	1.97E-56	8.22E-62	1	1.40E-48	2.81E-97	1.35E-87	2.29E-89
"	4.43E-87	5.47E-78	1.03E-192	8.24E-64	3.12E-73	1	1.72E-51	1.09E-84	1.45E-79	8.05E-98
"	8.35E-98	2.71E-89	5.68E-176	5.79E-70	3.19E-58	1	4.07E-48	1.50E-104	8.73E-128	3.34E-87
"	1.51E-85	9.47E-69	6.08E-168	7.91E-53	2.99E-66	1	1.55E-48	3.43E-81	2.08E-74	2.12E-91
"	1.04E-92	2.80E-71	1.45E-174	1.08E-67	1.12E-87	1	9.97E-86	4.52E-117	1.70E-125	5.20E-129
L. welshimeri WSLC 50146	8.83E-34	2.04E-50	6.94E-300	6.68E-32	7.97E-08	4.95E-58	0.99	6.07E-74	4.21E-166	1.34E-10
"	5.35E-33	2.35E-61	0	5.85E-46	2.10E-05	1.13E-68	0.999	2.22E-73	4.17E-220	1.23E-05
"	3.53E-33	1.69E-40	1.19E-299	1.14E-26	1.78E-07	2.04E-48	0.999	3.62E-72	1.58E-154	4.67E-13
"	9.69E-35	1.42E-43	0	2.50E-31	0.0002	7.51E-60	0.999	3.46E-82	1.14E-165	3.92E-07
"	5.19E-37	6.73E-51	8.49E-307	1.51E-39	2.41E-09	1.33E-45	0.999	6.29E-69	7.07E-153	1.41E-11

L. welshimeri WSLC 50150	1.64E-37	6.92E-121	6.38E-248	5.67E-114	7.84E-104	5.88E-98	6.06E-69	1	1.28E-277	9.26E-97
“	7.64E-68	2.08E-170	1.07E-228	5.97E-164	3.31E-167	7.60E-122	5.68E-123	1	3.29E-312	2.30E-157
“	2.20E-37	2.62E-109	1.03E-219	8.75E-102	1.77E-104	3.66E-88	2.13E-67	1	8.09E-244	2.04E-94
“	7.28E-31	2.03E-110	6.55E-222	2.52E-102	8.71E-100	1.91E-86	5.76E-65	1	7.81E-248	9.75E-89
“	3.77E-34	3.43E-117	2.87E-248	2.16E-106	1.17E-100	1.09E-99	5.09E-68	1	5.26E-249	5.92E-84
L. innocua ATCC 33090	7.87E-229	1.04E-153	6.32E-305	4.22E-131	1.86E-185	2.35E-81	7.81E-152	1.24E-243	1	1.81E-213
“	2.60E-286	2.72E-217	0	5.21E-184	1.78E-224	4.61E-102	1.08E-187	1.19E-280	1	1.29E-254
“	0	1.58E-224	0	3.72E-199	1.18E-280	7.70E-130	4.13E-235	0	1	2.05E-316
“	5.27E-248	1.13E-167	0	2.82E-146	9.97E-198	7.31E-95	3.98E-167	5.04E-275	1	9.42E-227
“	2.21E-273	1.66E-175	4.66E-287	3.94E-133	2.23E-180	6.33E-65	7.85E-143	2.67E-293	1	2.13E-218
L. ivanovii ATCC 19119	9.91E-36	2.02E-59	0	3.21E-50	7.19E-08	7.15E-98	1.16E-07	1.37E-89	1.00E-236	0.999
“	3.34E-35	2.45E-57	0	2.02E-47	0.0001	3.50E-94	6.70E-06	3.48E-91	1.76E-228	0.999
“	7.56E-46	4.79E-72	0	4.82E-63	1.83E-09	3.25E-83	6.79E-07	1.76E-86	1.83E-209	0.999
“	3.58E-59	2.35E-74	0	8.12E-65	1.92E-09	8.90E-110	3.54E-14	2.01E-123	1.24E-229	0.999
“	6.71E-60	8.41E-79	0	5.57E-66	9.71E-11	1.30E-106	6.68E-11	1.22E-116	6.34E-220	1

Supplementary Table 7 Fatty acid masses utilized for multivariate analysis

**Fatty acid**	**Mass**	**[M-H]$^-$**
C14:1	226.2	225.2
C14:0	228.2	227.2
C15:0	242.2	241.2
C16:1	254.2	253.2
C16:0	256.2	255.2
C17:1	268.2	267.2
C17:0	270.3	269.3
C18:2	280.2	279.2
C18:1	282.3	281.3
C18:0	284.3	283.3
C19:0	298.3	297.3
C20:0	312.3	311.3
C21:0	326.3	325.3
C22:1	338.3	337.3
C22:0	340.3	339.3
C23:1	352.4	351.4
C23:0	354.3	353.3
C24:1	366.3	365.3
C24:0	368.4	367.4
C25:1	380.4	379.4
C25:0	382.4	381.4
C26:1	394.4	393.4
C26:0	396.4	395.4
